# The use of interventional procedures for cancer pain. A brief review

**DOI:** 10.1007/s00520-024-08467-6

**Published:** 2024-04-12

**Authors:** Yi-Ching Lee, Timothy Brake, Emma Zhao, Alix Dumitrescu, Wei Lee, Benjamin Tassie, Kok-Eng Khor, Andy Yi-Yang Wang

**Affiliations:** 1 Department of Anaesthetics and Pain Management Centre, Royal Prince Alfred Hospital, Level 4, QEII, Missenden Rd, Camperdown, Sydney, NSW 2050 Australia; 2 Department of Anaesthetics and Pain Service, Chris O’Brien Lifehouse, Sydney, NSW Australia; 3 School of Medicine, Faculty of Medicine & Health, University of Sydney, Sydney, NSW Australia; 4 Improving Palliative, Aged and Chronic Care through Clinical Research and Translation (IMPACCT), Faculty of Health, University of Technology Sydney, Sydney, NSW Australia; 5 Sydney Nursing School, Faculty of Medicine & Health, University of Sydney, Sydney, NSW Australia; 6 Department of Palliative Medicine, Royal Prince Alfred Hospital, Sydney, NSW Australia; 7 HammondCare, Royal North Shore Hospital, Sydney, NSW Australia; 8 Pain Management Department, Prince of Wales Hospital, Sydney, NSW Australia; 9 Northern Clinical School, University of Sydney, St Leonards, Australia

**Keywords:** Cancer-related pain, Pain management, Cordotomy, Peripheral nerve blocks, Neuraxial analgesia, Sympathetic blocks

## Abstract

**Context:**

Pain is a common experience in people living with cancer. Concerns around opioid prescribing have seen a move toward a multi-modality management approach, which includes interventional pain procedures.

**Purpose:**

In this paper we discuss the interventional pain procedures used to treat cancer pain at two major tertiary centers in Australia.

**Methods and results:**

This expert review provides practical insights on cancer pain management from healthcare providers in different specialties. These insights can be used to guide the management of a wide range of cancer pain types.

**Conclusions:**

Furthermore, this review identifies the need for a systematic and comprehensive approach to the management of cancer pain that is broader than that of a single specialty. With recent advances in pain management procedures, an interdisciplinary approach is essential in order to provide an up to date, patient tailored approach to pain management.

This review will help inform the development of a cancer pain intervention registry.

## Introduction

### Cancer pain

Pain is a common experience in people living with cancer; up to 80% of patients with advanced cancer experience pain [1]. Studies showed that more than one third of individuals report moderate to severe pain and up to 10% of patients experience chronic severe pain [2]. Cancer pain can be attributed to the malignancy, its complications, or side effects of treatments (e.g., post-surgical pain and chemotherapy-induced peripheral neuropathy) [3, 4]. The underlying pathogenesis may involve not only the nociceptive mechanism caused by ongoing tissue damage, but also neuropathic and sensitization processes caused by atypical somatosensory processing in the peripheral or central nervous systems [4, 5].

**Fig. 1:**
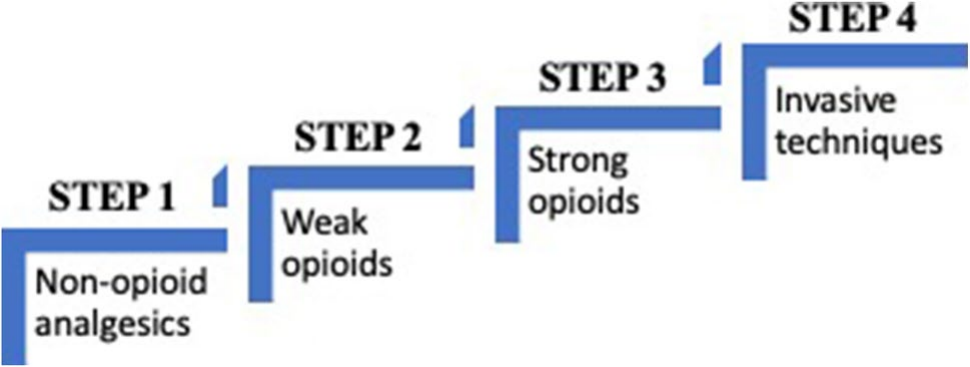
A representation of Vargas-Schaffer adaptation of the WHO analgesic ladder

In 1985, the World Health Organization proposed the analgesic ladder [6], which supported commencing the treatment of cancer pain with non-opioid medications, before progressing to weak opioids followed lastly by strong opioids. However, both the opioid crisis and the fact that patients can become unresponsive to strong opioids necessitated the re-examination of the use of opioids in the treatment of pain, including cancer pain [7, 8]. The analgesic ladder has subsequently been modified over time, with Vargas-Schaffer suggesting the inclusion of a fourth step which added invasive techniques to the pain management armament (Fig. 1) [8, 9].

With both advances in imaging technology and a better understanding of cancer pain pathogenesis, there has been growing utilization of interventional techniques in the management of cancer pain. The use of advanced imaging modalities has facilitated more and more accurate target localization with less invasive techniques than in the past. In the last few decades, interventions relied initially on landmarks and later on two-dimensional fluoroscopy with X-ray guidance. With growing availability and portability of CT scans, much better precision is achievable with three-dimensional real-time visualization. It also further facilitates treatment of smaller structures such as spinal nerve targets and allows better localization of structures in those with difficult anatomy due to prior surgical procedures or presence of malignancy distorting normal anatomy.

## Pain prevention

All patients undergoing cancer treatment should undergo prehabilitation strategies, where possible [10]. Total body prehabilitation can improve post-operative pain. For example, psychological prehabilitation prior to surgery can reduce post-operative pain in people undergoing breast surgery [11], and pre-operative exercise programs in this setting may also reduce post operative pain [12].

## Interventional pain procedures

Interventional pain procedures refer to treatments directly applied on a neuraxial region, autonomic nerve plexuses, or peripheral nerves [13]. This may involve the delivery of a drug (often with local anesthetic or neurolytic agents), modulation of nerve transmission through electrical impulses with radiofrequency ablation device or use of spinal cord stimulator, or destructive procedures such as cordotomy [13, 14]. However, apart from celiac plexus blocks, high-quality evidence supporting the use of most of these procedures is lacking, with most evidence derived from retrospective studies or case series conducted in single health institution [15–18].

Here we explore the range of interventional procedures performed in two major cancer centers in New South Wales, Australia. The interventional pain procedures used to treat cancer pain are outlined in Table 1.

**Table 1:** Potential categories for intervention

Nerve block and neurolysis^a^	
Peripheral nerve	
Nerve plexus	
Neuraxial -Epidural and intrathecal interventions (e.g. saddle block) -Percutaneous cervical cordotomy	
Implantable devices	
Targeted drug delivery (e.g. intrathecal pump)	
Nerve stimulators (targeting spinal cord and/or peripheral nerve)	
Surgical options (minimally invasive and open techniques)	
Bilateral open thoracic cordotomies	
Dorsal root entry zone lesioning procedures	
Radiological options	
Vertebroplasty	
Emerging options	
Percutaneous trans-arterial embolization	

^a^Nerve blocks use local anesthetic solutions and have a temporary effect; neurolysis uses destructive agents such as alcohol or phenol to destroy the affected tissues and nervous structures

### Peripheral nerve blocks

Peripheral nerve blocks work by interrupting nociceptive transmission from a regional nerve innervating the painful structure by use of a local anesthetic injection. Common applications include intercostal nerve blocks for rib pain from bony metastasis, trigeminal nerve blocks for head and neck malignancy, femoral/sciatic nerve blocks for lower limb pain, or serratus anterior blocks for chest wall pain following mastectomy or thoracic surgery. For longer duration of effect lasting from days to weeks, an indwelling catheter for repeated bolus doses or continuous infusion may be considered.

In patients with progressive pain, neurolytic blocks with injection of alcohol or phenol are often the intervention of choice for their more definitively destructive mechanism on affected tissues and nervous structures [19, 20]. The main concerns of peripheral nerve neurolysis include neuritis, painful dysesthesia, or deafferentation pain worsening existing symptoms, and motor weakness or bowel/bladder incontinence if motor/autonomic nerves are involved [19, 20]. The effectiveness and rate of adverse events with peripheral nerve intervention in cancer pain management require further evaluation as most evidence has been derived from case series [19, 20].

### Nerve plexus/sympathetic blocks

Interruption of afferent or sympathetic nerve pathways forms the basis for analgesia in relevant body parts. Visceral afferent nociception from cancer involving upper abdominal structures such as the pancreas, liver, stomach, adrenals and kidneys travels through the celiac plexus and then through the splanchnic nerves to the central nervous system.

For lower abdominal and pelvic structures such as the bladder, prostate, sigmoid colon, uterus, ovaries, vaginal fundus, and rectum, the afferent nociceptive signals pass through the superior hypogastric plexus. For perineal structures such as the anus, distal rectum, urethra, scrotum, vulva, and vagina, afferent signals pass through the ganglion impar (ganglion of Walther) located on the anterior surface of the sacrum at the sacrococcygeal junction [21].

**Fig. 2:**
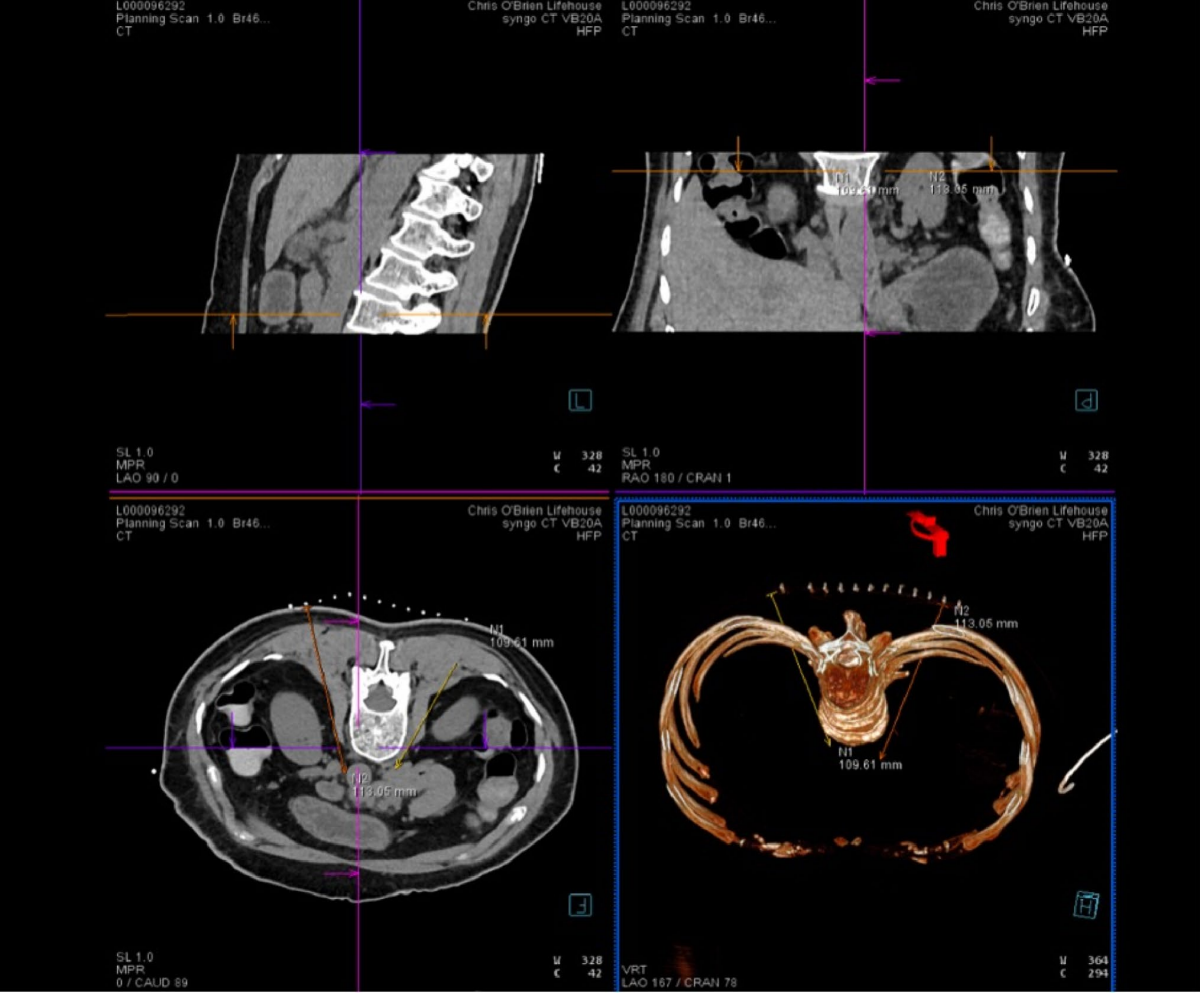
The planning CT scan provides 3-D visualization before needle placement in a patient with advanced pancreatic cancer

**Fig. 3:**
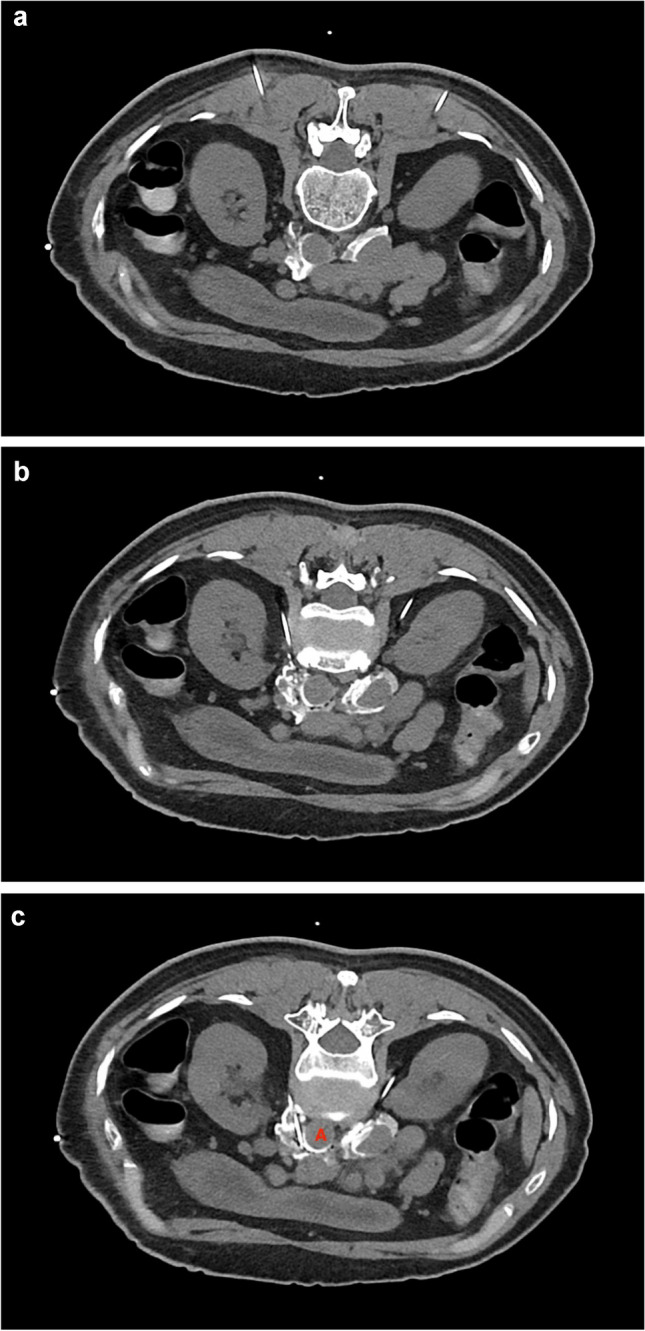
Real-time visualization of both needles as they are advanced in a patient with advanced pancreatic cancer. A aorta

Among cancer pain interventions, celiac plexus block and neurolysis have been most heavily studied, and their use has been largely supported by randomized controlled trials and systematic review for analgesic efficacy [22, 23]. Most of the evidence, however, draws on the early studies using a percutaneous fluoroscopy guided approach. Later studies demonstrated non-inferiority from ultrasound-guided modality [24]. Advancing techniques has seen the increasing utilization of CT guided approach to provide three-dimensional visualization of needle placement [25] (Figs. 2 and 3). These advances necessitate the involvement of an interventional radiologist in the interdisciplinary team.

**Fig. 4:**
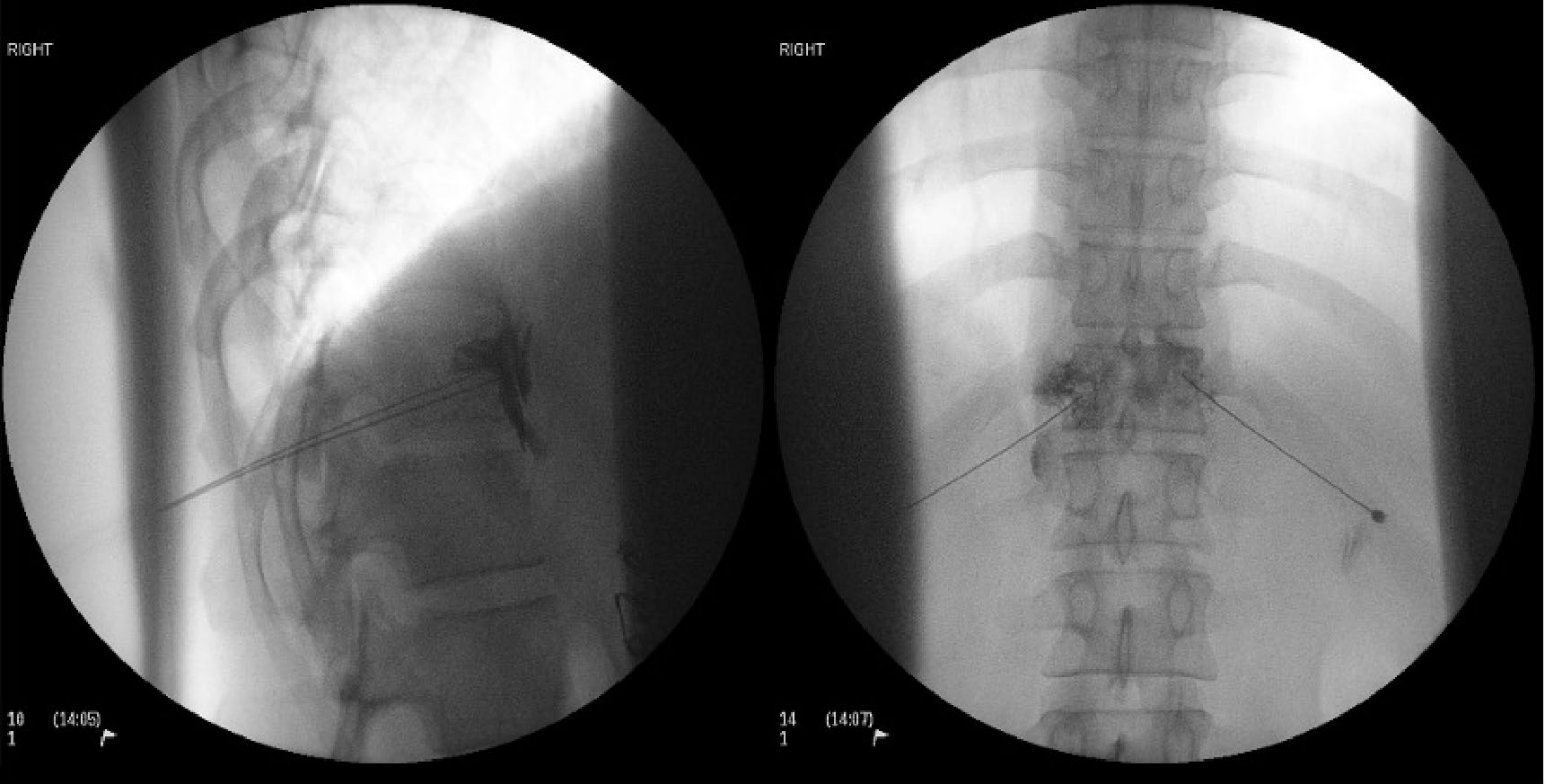
Images from splanchnic nerve block with fluoroscopy/Xray guidance

Other sympathetic blocks that can be performed include stellate ganglion blocks for managing pain in the head and neck, breast, and upper limb region and also lumbar sympathetic blocks for sympathetically maintained lower limb pain [26, 27]. Similarly, randomized controlled studies that assess these are lacking in the literature. Splanic nerve block with fluoroscopy/X-ray guidance is shown in  Fig. 4.

### Intrathecal analgesia

Intrathecal analgesia involves the delivery of medication into cerebrospinal fluid in the vicinity of the intended spinal level/s . Intrathecal drug delivery has been increasingly utilized over the last few decades in patients with cancer that does not respond to conventional medical management or where there are intolerable side-effects systemic analgesic agents. The use of intrathecal opioid analgesia is well supported by reviews on safety, pain reduction, and cost-effectiveness and may be supplemented with other agents off label including local anesthetics or clonidine [28, 29].

The drug/s may be delivered through a single bolus injection, through external infusion pump, or a fully implantable device. The choice of delivery method depends largely on the clinical situation and local nursing expertise and availability. External infusion pumps are usually considered for patients with a limited prognosis measured in weeks, whereas fully implantable devices are more appropriate for patients with a life expectancy greater than 6 months [28, 29].

For cancer patients with poor prognosis and poor functional status, a saddle block, involving administration of neurolytic agents such as alcohol or phenol, can be a helpful procedure for perineal pain. It produces a more prolonged clinical effect at the expense of motor and autonomic functions [28, 29]. Careful patient positioning by an experienced interventionalist is essential, for example sitting with a slight backwards lean when using heavy phenol or in the prone Trendelenburg position when using hypobaric alcohol to avoid spreading to adjacent sensorimotor nerves.

The other potential intervention includes use of a spinal cord stimulator by placement of an electrical lead in the epidural space [30]. However, apart from an associated reduction in opioid use, there is very limited evidence of effectiveness in the management of cancer pain [30].

### Cordotomy

Cordotomy is an ablative procedure of the spinal cord (predominately the spinothalamic tract) performed almost exclusively for cancer-related pain. Unilateral percutaneous cordotomy with entry at the level of C1/2 on the contralateral side to the pain is the most common approach, although it can also be performed endoscopically, transdiscally, or as an open procedure [31].

**Fig. 5:**
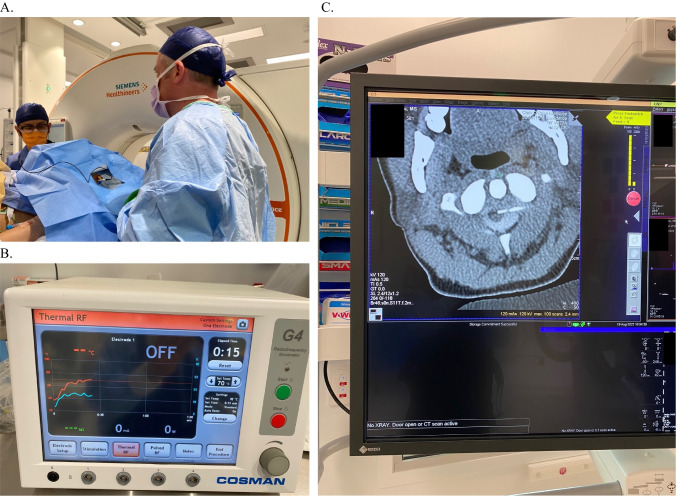
Percutaneous cordotomy procedure for treatment of right hip and leg pain caused by metastatic NSCLC (non-small cell lung cancer) in the pelvic region. **A** A needle with pulse generator is placed over left neck region for real-time sensory testing to precisely localise the area of pain with CT image guidance. **B** Radiofrequency device providing thermal lesioning of the spinothalamic fibers. **C** CT image inside theatre guiding needle placement through C1/2 vertebral space into targeted spinal cord

Percutaneous cordotomy requires the patient to be awake and cooperative to allow for sensory stimulation and feedback, whereas the open procedure is done under general anesthesia (Fig. 5). Percutaneous cordotomy is indicated for those with poorly controlled unilateral pain (below the level of the C4 dermatome) and with an estimated prognosis of less than 6 to 12 months [32]. The overall complication rates with CT guidance are low, with severe respiratory compromise and motor weakness being the major concerns.

For those with axial pain below the diaphragm, a bilateral thoracic cordotomy is more appropriate [18]. This requires the expertise of neurosurgeons as an open procedure. Similarly, its use is supported by many case series [33, 34].

### Radiofrequency ablation

Radiofrequency ablation is a high temperature thermal therapy that induces coagulation necrosis [35]. It is performed under image guidance and is a useful treatment particularly for patients with painful vertebral metastases [36].

### Vertebroplasty

Vertebroplasty involves the injection of artificial bone cement and an opacifier into the inter-trabecular marrow space of fractured vertebrae [37]. It is performed with imaging guidance under local anesthesia [37, 38]. It is useful for painful vertebral compression fractures caused by metastases or multiple myeloma [37, 38] and can be used in patients receiving adjuvant radiation, surgical therapy, or chemotherapy [37]. In Australia, vertebroplasty is performed infrequently due to its invasive nature and lack of clear evidence of its benefit.

### Trans-arterial embolization

Trans-arterial embolization involves the injection of embolic agents in the arteries supplying the tumor and is well known for management of spinal bone lesions. There is emerging evidence, primarily based on retrospective studies, demonstrating the potential benefits for treatment of painful bony metastases refractory to conventional radiotherapy [39, 40].

## Barriers to cancer pain management

It has long been recognized that cancer pain is undertreated, despite the availability of evidence-based guidelines. An Australian survey of palliative care specialists (*n* = 92) identified the following barriers to the provision of pain management: insufficient access to nonpharmacologic interventions, patient comorbidities, and a lack of coordination between services [41]. A systematic review of service delivery models for cancer pain recommended the development of policies for referrals to a pain consultation service which can be integrated into a clinical pathway [42]. This would help to address the lack of coordination between services.

A recent qualitative study to identify barriers and facilitators of interventional management of cancer pain interviewed healthcare professionals (*n*=24) involved in cancer care at two cancer centers in Sydney (unpublished). The study identified six themes, which overlapped with the previous findings. These were (1) clinicians’ knowledge and awareness of interventional pain management, (2) training and resources on interventional pain procedures, (3) unclear referral pathways, (4) clinician perceptions of safety and efficacy, (5) the timing of the intervention, and (6) the need for holistic healthcare. Anecdotally, clinical practices appear to vary significantly due to different expertise and institutional preferences.

A broader review of barriers to optimal cancer pain management identified not only clinician barriers but also society’s attitude toward pain management, system barriers, patient barriers, and healthcare disparities [43]. With regard to healthcare disparities, we know that cancer incidence is higher in New South Wales, Australia outside of the major cities, of which Sydney is one [44]. It is highly likely that access to interventional pain management approaches are limited for patients living with cancer in regional and remote parts of Australia.

Another older Australian study explored patient barriers to optimal pain management. It identified three barriers: (1) poor levels of patient knowledge about pain, (2) low perceived control over pain, and (3) a lack of communication about pain [45]. Without knowing more about how cancer pain is currently being managed it is hard to suggest strategies to overcome these barriers. However, patient education about pain management should not be discounted.

## Chronic pain

As cancer survivorship improves, it is important to be aware of the possibility of chronic pain. Cancer survivors often experience adverse physical and psychosocial effects from the diagnosis and its treatment, and should not be overlooked when taking a chronic pain management approach [46, 47]. Cancer survivors with chronic pain also report lack of sleep, fatigue, and mental health issues [47]. In our experience some cancer survival present for repeated interventional pain management procedures, for example, intercostal radiofrequency ablation for persistent post-mastectomy, or post sternotomy pain [48]. Cancer survivors should be assessed and be appropriately referred for treatment of chronic pain, where appropriate. Currently there is very limited evidence to specifically address chronic pain issues in this unique population, and more research into effective strategies is needed.

## The need for a cancer pain intervention registry

This review shows the wide range of pain intervention procedures that are currently used in the management of cancer pain. However, to date, there is deficiency of evidence in specific applications, indications, efficacy, or adverse events for varying interventions in treatment of cancer pain. The most recent cancer incidence data for New South Wales, Australia showed 48,165 new cancer diagnoses in 2020 [49]. People are living with cancer for longer than ever before [50], and over one third of cancer patients report moderate to severe cancer pain [2], so the need to understand best practices in cancer pain management is growing.

The establishment of a national cancer pain intervention registry should be considered. This would provide insight into the different interventional pain procedures used in patients with cancer, how these procedures are accessed, their perceived efficacy, and geographical variation. Such a registry would also capture information about the roles and involvement of the interdisciplinary team in the successful management of cancer pain.

## Conclusions

This review has provided an overview of interventions used in the management of cancer pain through leading clinicians in the field of pain management and palliative care. It identified need for better evidence guiding future practice, in particular, through establishment of an intervention registry. It also pointed out barriers in achieving optimal pain management, which included lack of co-ordination of suitable interventional pain management in the heterogenous cancer population as one major issue.

## Data Availability

No datasets were generated or analysed during the current study.
